# Aggression and its association with suicidality in migraine patients: a case-control study

**DOI:** 10.1186/s10194-018-0903-x

**Published:** 2018-08-14

**Authors:** Sung-Pa Park, Jong-Geun Seo

**Affiliations:** Department of Neurology, School of Medicine, Kyungpook National University, Daegu, Republic of Korea

**Keywords:** Aggression, Migraine, Suicidality, Determinant, Anxiety, Headache intensity

## Abstract

**Background:**

To identify aggression and its association with suicidality in migraine patients.

**Methods:**

We enrolled 144 migraine patients who made their first visit to our headache clinic. We collected data regarding their clinical characteristics and the patients completes the Aggression Questionnaire (AQ) and other questionnaires. We also interviewed patients with the Mini International Neuropsychiatric Interview—Plus Version 5.0.0 (MINI) to identify their suicidality. The degree of aggression in migraine patients was compared to the degree of aggression in healthy controls. Major determinants for aggression and its association with suicidality were also examined.

**Results:**

The overall AQ score and anger and hostility subscale scores were higher in migraine patients than controls. For migraine chronicity, patients with chronic migraine (CM) had a higher overall AQ score and physical aggression, anger, and hostility subscale scores than controls. On the other hand, all AQ scores in patients with episodic migraine were not different from the scores of the controls. Although several factors were associated with the overall AQ score, major determinants were anxiety (ß = 0.395, *p* < 0.001), headache intensity (ß = 0.180, *p* = 0.016), and CM (ß = − 0.165, *p* = 0.037). Patients who had suicidality based on the MINI showed a higher overall AQ score than patients without suicidality (*p* < 0.001).

**Conclusions:**

Aggression is likely to be a common feature in CM. Comorbid aggression may help to identify suicidality in migraine patients.

## Background

Migraine is a disabling neurological disorder due to recurrent attacks of headache and accompanying psychosomatic symptoms such as depression, anxiety, sleep problems, and fatigue [1–3]. These symptoms restrict daily activities, heighten mortality, impair quality of life and increase the social burden [1, 3, 4]. Migraine was a main cause of burden for the Korean public in a review study on global burden of disease in 2013 [5]. For these reasons, migraine patients should be managed appropriately to lessen those problems.

Aggression is a psychobehavioral symptom defined as overt, often harmful, social interaction that is intended to inflict damage or other unpleasantness on another people [6]. It places substantial burden and costs on human society [7]. Aggression takes a variety of forms including aggression-related feelings, such as anger or hostility, and aggression-related behaviors such as physical or verbal aggression [8]. Data from human and animal studies suggested that the amygdala and associated limbic structures, the frontal lobes, the periaqueductal gray, and the hypothalamus were responsible for the manifestation of aggression [9]. Aggressive behaviors have been commonly reported in psychiatric disorders, such as intermittent explosive disorder, psychosis, and substance abuse, and in neurological disorders, such as epilepsy and Alzheimer’s disease [10].

Migraine patients have been reported to exhibit aggressive behavior as a specific personality traits, along with neuroticism and borderline personality disorder (BPD) [11]. Migraine patients showing neuroticism were more likely to experience depression, anxiety, fear, anger, frustration, and envy than ordinary people [12]. Moreover, patients with a high level of neuroticism were at risk for the development of psychiatric disorders [11]. The hallmark features of BPD are fluctuating instability in self-image, interpersonal relationships, and emotions [13]. Intense mood swings, impulsive behaviors, and extreme reactions can make it difficult for people with BPD to complete schooling, maintain stable jobs and have long-lasting, healthy relationships. Approximately, 10% of people affected by BPD kill themselves [13]. Due to these reasons, the existence of aggression is likely to be an important issue in migraine patients. However, the nature of aggression in migraine patients has been underrecognized and has not been examined systematically.

Aggression occurs in relation to physical pain. In the General Aggression Model, physical pain as a situational factor influences cognitions, feelings, and arousal, which in turn affects appraisal and decision processes, which in turn influence aggressive or nonaggressive behavioral outcomes [14]. Migraine patients suffer from recurrent severe headache as physical pain. In this context, it is important to understand the appearance of aggression in relation to the appearance of a headache. Aggression is a facilitator for aggravating suicidality levels up to highly lethal suicidal attempts in the atmosphere of unbearable mental pain including depression, anxiety, hopelessness, and distress [15]. Migraine has been known to be associated with depression, anxiety, and suicidality [2, 16]. Therefore, it is also important to acknowledge aggression in relation to suicidality in migraine patients because lethal suicidal attempts are associated with mortality. Therefore, the aim of our study is to investigate the clinical significance of aggression and its association with suicidality in migraine patients.

## Methods

### Subjects

New patients with migraine who consecutively visited our headache clinic were enrolled from January 2017 to September 2017. Patients aged between 20 and 70 were included. Patients were diagnosed by the International Classification of Headache Disorders 3rd edition, beta version [17]. Patients who had illiteracy, mental retardation, serious medical, neurological, or psychiatric disorders, and history of alcohol or drug abuse that prevented them from cooperating with us during the study were excluded. Patients who refused to complete questionnaires and whose diagnosis was a probable migraine were also excluded. Initially, 163 new patients visited our clinic. Of them, 19 patients were excluded due to a probable migraine diagnosis (*n* = 8), refusal to enter the study (*n* = 7), younger age (*n* = 3), and illiteracy (*n* = 1). In the end, 144 patients were eligible for the study. The same number of age-, gender-, and education-matched healthy controls were also invited to participate. They were university students, office workers, teachers, or hospital employees.

### Study design

This is a case-control study. The Institutional Review Board of Kyungpook National University Hospital approved the study. All participants gave written informed consent. SP Park interviewed each patient and collected demographic and clinical information. We collected data on their employment and household income, height and weight, age at onset of migraine, duration of migraine, type of migraine, migraine chronicity, family history of migraine, medication overuse headache (MOH), headache intensity, and associated symptoms including nausea and/or vomiting, photophobia, phonophobia, osmophobia, and allodynia. Body mass index (BMI) was calculated from height and weight. Headache intensity was measured by a visual analog scale (VAS). Patients were asked to describe the highest intensity of headache in the preceding 4 weeks. Photophobia, phonophobia, and osmophobia during migraine attacks were defined as hypersensitivity to light, sound, and certain odors, respectively. Allodynia was measured by the 12-item Allodynia Symptom Checklist (ASC-12) with a cut-off score of > 2 to define allodynic patients [18].

Eligible subjects completed several self-reported questionnaires, including the Aggression Questionnaire (AQ) [19], the Migraine Disability Assessment Scale (MIDAS) [20], the Patient Health Questionnaire-9 (PHQ-9) [21], the Generalized Anxiety Disorder-7 (GAD-7) [22], the Epworth Sleepiness Scale (ESS) [23], and the Insomnia Severity Index (ISI) [24]. A trained neuropsychologist assessed the suicidality of patients with the suicidality module of the Mini International Neuropsychiatric Interview—Plus Version 5.0.0 (MINI) [25].

### Questionnaires

#### Aggression questionnaire (AQ)

The AQ was developed by Buss and Perry; it measures aggressive behavior and consists of 29 items in four subscales: Physical Aggression (9 items), Verbal Aggression (5 items), Anger (7 items) and Hostility (8 items) [26]. Responses to all items are given on a five-point Likert scale ranging from ‘never’ (1) to ‘always’ (5); subscale scores can be summed to obtain an overall score. Higher scores indicate greater aggression. A validated Korean version of the AQ has been produced [19]. During the validation process, a decision was taken to omit two of the original items from the anger subscale (‘Some of my friends think I’m a hothead’ and ‘Sometimes I fly off the handle for no good reason’) because they related more to verbal aggression and hostility than anger. For this study, 27 items were used for the evaluation of aggression. Cronbach’s a coefficient for the Korean version of the AQ was 0.86.

#### Migraine disability assessment scale (MIDAS)

The Korean version of the MIDAS includes a 5-item questionnaire to evaluate disability during the past three months [20]. Scores were used to measure the overall level of disability. Cronbach’s α coefficient of this scale was 0.75.

#### Patient health Questionnaire-9 (PHQ-9)

The Korean version of the PHQ-9 has been validated in patients with migraine [21]. It includes nine items pertaining to DSM-IV criteria for major depressive disorder (MDD). The overall score ranges from 0 to 27, with a higher score indicating a higher degree of depressive symptoms. Cronbach’s α coefficient of this scale was 0.894.

#### Generalized anxiety Disorder-7 (GAD-7)

The Korean version of the GAD-7 has been validated in patients with migraine [22]. It consists of seven items pertaining to DSM-IV criteria for GAD. The overall score ranges from 0 to 21, with a higher score meaning a higher degree of anxiety symptoms. Cronbach’s α coefficient of this scale was 0.915.

#### Epworth sleepiness scale (ESS)

The Korean version of the ESS has been validated in patients with obstructive sleep apnea [23]. ESS is composed of eight questions, each of them asking about the subject’s likelihood of dozing off or falling asleep in a particular situation that is common in daily life. A higher score indicates a higher subjective sleepiness. Cronbach’s α coefficient of ESS was 0.917.

#### Insomnia severity index (ISI)

The Korean version of the ISI has been validated in patients with sleep disorders [24]. The ISI is a seven-item questionnaire that measures patient’s perception of insomnia severity. Its total score ranges from 0 to 28, with a higher score indicating a greater insomnia severity. Cronbach’s α coefficient of ISI was 0.92.

### Interview

#### MINI international neuropsychiatric interview—Plus version 5.0.0 (MINI)

The MINI is a brief, structured interview based on DSM-IV criteria [25]. For identifying suicidality, a neuropsychologist (JH Lee) conducted the suicidality module of the MINI. It is composed of six questions that are weighted differently, and include five questions asking about current suicidality (which includes suicidal ideation or an attempt in the preceding month; the questions were about a wish for death [weight 1], a wish for self-harm [weight 2], suicidal thoughts [weight 6], a suicide plan [weight 10], and a suicide attempt [weight 10], and one question asking about lifetime suicide attempts [weight 4]). If respondents said “yes” for at least one of the six questions, they were thought to have suicidality. The degree of current suicidality was estimated from the sum of the weighted score of the six questions, with low (1–5), moderate (6–9), and high (≥10) levels of suicide risk.

### Statistical analyses

Statistical Package for Social Sciences (SPSS version 22.0) was used for data analysis. Descriptive statistics are presented as counts, percentages, means, and standard deviations. Student’s *t*-test or chi-squared test was applied to compare two groups. To determine the relationship between various independent variables and the overall AQ score, Pearson’s correlation coefficient was determined. Dummy variables are used for categorial variables. Variables having significant correlation with the overall AQ score were then included in multiple linear regression analyses with stepwise selection using entry and exit probabilities of 0.05 and 0.1, respectively. Statistical significance was considered at *p* < 0.05.

## Results

Demographic, socioeconomic, clinical, and psychosomatic characteristics in migraine patients compared with healthy controls are listed in Table 1. Demographic and socioeconomic states and BMI in migraine patients were not different from those of healthy controls. Of 144 migraine patients, 56 patients (38.8%) had chronic migraine (CM) and 35 patients (24.3%) had suicidality.

**Table 1:** Characteristics of migraine patients and healthy controls

Characteristics	Mean ± SD (range) or number (%)		*P* value^*^
	Migraine patients	Healthy controls	
	(*n* = 144)	(*n* = 144)	
Age, years	37.5 ± 13.2 (20–64)	38.2 ± 12.0 (20–64)	0.642
Gender, female	117 (81.3)	115 (79.9)	0.882
Education, years	13.8 ± 2.5 (6–18)	14.1 ± 2.3 (6–18)	0.242
Employment, yes	63 (43.8)	74 (51.4)	0.238
Household income,	108 (75.0)	114 (79.2)	0.483
≥3 million KRW/month			
BMI	22.4 ± 3.4 (15–35)	22.5 ± 3.1 (15–33)	0.698
Age at onset, years	27.5 ± 11.2 (10–58)		
Disease duration, years	10.0 ± 8.5 (0.3–42)		
Type of migraine			
Migraine with aura	15 (10.4)		
Migraine without aura	129 (89.6)		
Migraine chronicity			
EM	88 (61.1)		
CM	56 (38.9)		
Family history of migraine	89 (61.8)		
MOH	11 (7.6)		
VAS	7.5 ± 2.3 (0–10)		
Associated symptoms			
Nausea/vomiting	121 (84.0)		
Photophobia	69 (47.9)		
Phonophobia	82 (56.9)		
Osmophobia	73 (50.7)		
Allodynia	29 (20.1)		
MIDAS, days	23.4 ± 26.3 (0–120)		
PHQ-9	6.0 ± 4.9 (0–24)		
GAD-7	4.6 ± 4.5 (0–21)		
ESS	5.6 ± 4.0 (0–20)		
ISI	8.1 ± 6.0 (0–26)		
MINI, suicidality	35 (24.3)		

*KRW* Korean Won, *BMI* Body Mass Index, *EM* episodic migraine, *CM* chronic migraine, *MOH* medication overuse headache, *VAS* visual analog scale, *MIDAS* Migraine Disability Assessment Scale, *PHQ-9* Patient Health Questionnaire-9, *GAD-7* Generalized Anxiety Disorder-7, *ESS* Epworth Sleepiness Scale, *ISI* Insomnia Severity Index, *MINI* Mini International Neuropsychiatric Interview—Plus Version 5.0.0

^*^Student’s t-test or chi-squared test was applied

The degree of aggression in migraine patients compared with healthy controls is documented in Table 2. Mean scores of the overall AQ, anger, and hostility were significantly higher in migraine patients than the score of the controls (*p* < 0.05 for the overall AQ score, *p* < 0.01 for anger and hostility). In CM patients, mean scores of the overall AQ, physical aggression, anger, and hostility were significantly higher than the scores of the controls (*p* < 0.05 for physical aggression, *p* < 0.01 for the overall AQ score, anger and hostility). On the other hand, all scores of the AQ in episodic migraine (EM) patients were not different from the scores of the controls.

**Table 2:** Aggression in migraine patients compared with healthy controls

	Mean ± SD (range)			
	Migraine patients	EM	CM	Healthy controls
	(*n* = 144)	(*n* = 88)	(*n* = 56)	(*n* = 144)
Overall AQ	48.9 ± 12.6 (31–105)^a^	45.3 ± 10.3 (31–76)	54.6 ± 13.8 (33–105)^b^	45.8 ± 8.5 (27–71)
Physical aggression	13.6 ± 4.2 (9–33)	12.7 ± 3.2 (9–26)	15.2 ± 5.0 (9–33)^a^	13.6 ± 3.6 (9–25)
Verbal aggression	9.9 ± 3.2 (5–20)	9.6 ± 3.2 (5–19)	10.3 ± 3.1 (5–20)	9.9 ± 2.7 (5–20)
Anger	11.6 ± 4.0 (5–24)^b^	10.3 ± 3.3 (5–22)	13.6 ± 4.3 (6–24)^b^	10.2 ± 2.6 (5–16)
Hostility	13.7 ± 5.2 (8–35)^b^	12.7 ± 4.3 (8–30)	15.4 ± 6.0 (8–35)^b^	12.2 ± 2.7 (8–22)

*EM* episodic migraine, *CM* chronic migraine, *AQ* Aggression questionnaire

Student’s t-test was conducted for the comparison between migraine patients and controls, ^a^*P* < 0.05, ^b^*P* < 0.01

Factors associated with the overall AQ score by univariate analyses in migraine patients are described in Table 3. Patients with CM, a higher headache intensity, and a higher score on the PHQ-9, the GAD-7, the ESS, and the ISI were associated with higher overall AQ scores. Major determinants for the overall AQ score by multivariate analyses in migraine patients are shown in Table 4. The strongest determinants was anxiety (*ß* = 0.395, *p* < 0.001), followed by headache intensity (*ß* = 0.180, *p* = 0.016) and CM (*ß* = − 0.165, *p* = 0.037).

**Table 3:** Factors associated with the overall AQ score by univariate analyses in migraine patients

Variable	*P* value (*r*)^*^
Migraine chronicity	< 0.001 (− 0.363)
VAS	< 0.001 (0.303)
PHQ-9	< 0.001 (0.432)
GAD-7	< 0.001 (0.490)
ESS	0.001 (0.267)
ISI	< 0.001 (0.294)

*AQ* Aggression Questionnaire, *BMI* Body Mass Index, *MOH* medication overuse headache, *VAS* visual analog scale, *MIDAS* Migraine Disability Assessment Scale, *PHQ-9* Patient Health Questionnaire-9, *GAD-7* Generalized Anxiety Disorder-7, *ESS* Epworth Sleepiness Scale, *ISI* Insomnia Severity Index

*Pearson’s correlation was applied

**Table 4:** Major determinants for the overall AQ score by multivariate analyses in migraine patients

Variable	Standardized	*P* value	Collinearity	Adjusted
	coefficients (*ß*)		(VIF)	*R* ^*2*^
Constant		< 0.001		0.293
GAD-7	0.395	< 0.001	1.168	
VAS	0.180	0.016	1.103	
Migraine chronicity	−0.165	0.037	1.231	

*AQ* Aggression Questionnaire, *GAD-7* Generalized Anxiety Disorder-7, *VAS* visual analog scale

**Fig. 1:**
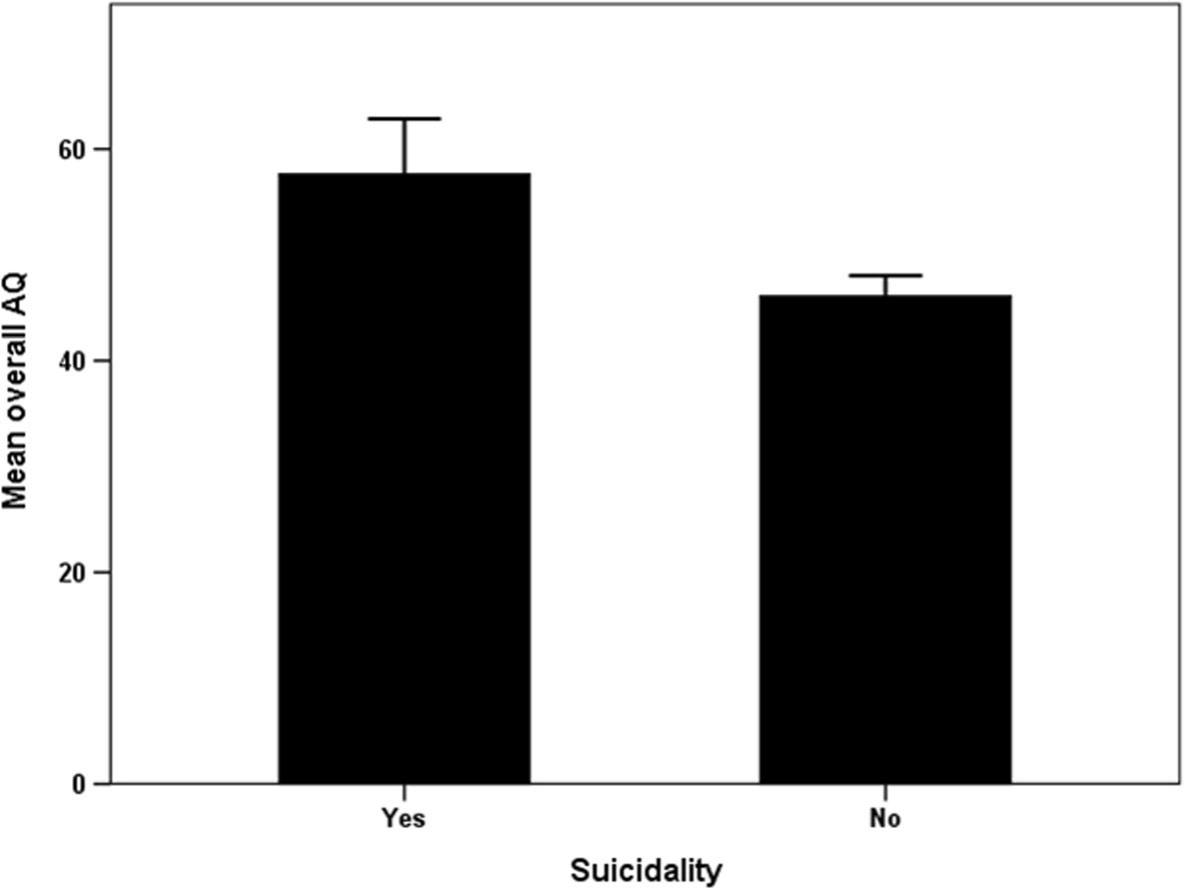
Comparison of the overall AQ score between patients having suicidality (*n* = 35) and those having no suicidality (*n* = 109). Mean score of the overall AQ was significantly higher in patients having suicidality than the mean score of patients having no suicidality (*p* < 0.001). Suicidality was determined by the MINI. Error bars represent 95% confidence intervals. AQ: Aggression Questionnaire, MINI: Mini International Neuropsychiatric Interview—Plus Version 5.0.0

Of patients showing suicidality (*n* = 35), 23 patients (68.6%) had a low level of suicide risk, 7 patients (20.0%) had a moderate level of suicide risk, and 4 patients (11.4%) had a high level of suicide risk. A lifetime suicide attempt was noted in 16 patients (45.7%). Regarding to migraine chronicity, the frequency of suicidality in CM patients (24 of 56 patients, 42.9%) was significantly higher than the frequency of suicidality in EM patients (11 of 88 patients, 12.5%) (*p* < 0.001). Mean scores of the overall AQ with respect to the existence of suicidality are illustrated in Fig. 1. Mean score of the overall AQ was significantly higher in patients having suicidality than patients with no suicidality (*p* < 0.001).

## Discussion

Although migraine seems to be associated with aggression, there are a few studies to examine the clinical significance of aggression in migraine patients. In a hospital-based study, migraine patients and tension-type headache (TTH) patients had a higher degree of hostility than healthy controls [27]. Migraine patients and TTH patients showed a significantly higher level of angry temperament and angry reaction [28]. Difficulty in anger management with a tendency to hypercontrol was shown in migraine patients compared with healthy controls [29]. In a multicenter study, self-aggression in migraine patients compared to healthy controls showed a trend towards higher scores due to a small sample size [30]. It was correlated with the level of headache-related disability. For CM patients, the frequency of passive-aggressive personality disorder was higher than the frequency of this disorder in healthy controls, and personality disorders were correlated with the MIDAS scores [31]. Taken together, these results seem to suggest that migraine patients are more likely to have aggression and to experience disability than healthy controls. Although we reported a higher level of aggression in migraine patients than controls, this appearance was confined to CM patients. Moreover, the degree of aggression was not correlated with the MIDAS score. Therefore, we suggest aggression may be a specific feature for CM regardless of headache-related disability.

We found CM, headache intensity, depression, anxiety, daytime somnolence, and insomnia as associated factors for aggression. However, major determinants for aggression were anxiety, headache intensity, and CM. That means depression and sleep problems are not critical for aggression after controlling for anxiety, headache intensity, and CM. It is well known that CM patients are more likely to have depression and sleep problems than EM patients [4, 32], and severe headache can elicit depression and sleep problems [33]. The reason why anxiety was the strongest factor for aggression may be attributed to a common pathology underlying anxiety and aggression. Amygdala function is known to play an important role in anxiety [34] and is also associated with emotional control processes and aggression [9]. Therefore, it is possible to suppose that anxious persons can also be aggressive. The contribution of headache intensity to aggression can be explained by the General Aggression Model [14]. We presume that recurrent severe headache as a situational factor is likely to induce aggression in migraine patients.

We do not consider anxiety and headache intensity as migraine-specific factors for aggression. These factors can be associated with aggression in other types of headache. For example, patients with cluster headaches and accompanying severe pain have been reported to have aggression [30]. On the other hand, migraine chronicity is likely to be a specific situation for provoking aggression in migraine patients. Recently, a high-resolution functional MRI study demonstrated that the hypothalamus played a crucial role in the pathophysiology of migraine chronification [35]. Because the hypothalamus is also an important pathogenic substrate for aggression, we presume aggression is likely to be a phenomenon of migraine chronicity. Further neuroimaging studies are needed to clarify the relationship between aggression and CM.

Migraine has been known to be associated with suicidality. In a systemic review of the literature, a modest positive association between migraine and suicidal ideation was found [16]. We also observed through psychiatric interviews, a high proportion (24.3%) of migraine patients had suicidal ideation or attempt. Because suicidality is closely related to mortality in mental disorders, it is important to identify risk factors for suicidality to lessen mortality. We found the overall AQ score was higher in patients with suicidality than those without suicidality. That means aggression may reflect suicidal ideation or attempt in migraine patients. Actually, in a genetic study of women with CM, those with affective temperaments including irritability were more likely to exhibit suicidal behavior [36]. Therefore, asking about aggressive feelings or behaviors in migraine patients will be another option for identifying suicidality, especially if clinicians feel uncomfortable asking them about suicidal ideation or attempts directly.

Our study has some limitations. First, subjects in a single tertiary hospital were investigated. Therefore, our results cannot be generalized. Second, this was a cross-sectional study. Causal relationships between variables could not be confirmed. We already presumed aggression was likely to be a phenomenon related to migraine chronicity due to sharing of pathologic substrates. However, it is also possible that aggression affected by recurrent migraine attack may induce functional changes of the brain and subsequently elicit CM. A longitudinal study is recommended to verify the causal relationship. Third, the suicidality module in the MINI includes questions about suicidal ideation and harming oneself. These do not always reflect actually committing suicide and increasing mortality. However, because aggression is a facilitator for aggravating suicidality levels to highly lethal suicidal attempts [15], further studies should be conducted to elucidate a role of aggression in the mortality of migraine patients.

## Conclusions

We found that the overall AQ score was higher in migraine patients than controls. Patients with CM had a higher overall AQ score and physical aggression, anger, and hostility subscale scores than controls. On the other hand, all AQ scores in patients with EM were not different from those of controls. Aggression is likely to be a common feature in CM. Major determinants for the overall AQ score were anxiety, headache intensity, and CM. Patients who had suicidality showed a higher overall AQ score than those without suicidality. Therefore, the recognition and proper management of aggression will be helpful for reducing mortality in CM patients.

## Abbreviations

AQ: Aggression Questionnaire
ASC-12: 12-item Allodynia Symptom Checklist
BMI: body mass index
BPD: borderline personality disorder
CM: chronic migraine
EM: episodic migraine
ESS: Epworth Sleepiness Scale
GAD: Generalized Anxiety Disorder
ISI: insomnia severity index
MDD: major depressive disorder
MIDAS: Migraine Disability Assessment Scale
MINI: Mini International Neuropsychiatric Interview
MOH: medication overuse headache
PHQ: Patient Health Questionnaire
TTH: tension-type headache
VAS: Visual Analog Scale
